# Spectra, intermittency, and extremes of weather, macroweather and climate

**DOI:** 10.1038/s41598-018-30829-4

**Published:** 2018-08-23

**Authors:** S. Lovejoy

**Affiliations:** Physics, McGill University, Montreal, Que H3A 2T8 Canada

## Abstract

It was recently found that the accepted picture of atmospheric variability was in error by a large factor. Rather than being dominated by a series of narrow scale-range quasi-oscillatory processes with an unimportant white noise “background”, it turned out that the variance was instead dominated by a few wide range scaling processes albeit occasionally interspersed with superposed quasi-oscillations. Although the classical model implied that successive million year global temperature averages would differ by mere micro Kelvins, the implausibility had not been noticed. In contrast, the new picture inverts the roles of background and foreground and involves four (possibly five) wide range scaling processes. As with any new paradigm, there are consequences; in this paper we focus on the implications for the spectra, intermittency and the extremes. Intermittency is an expression of the spatio-temporal sparseness of strong events whereas the extremes refer to the tails of their probability distributions and both affect the spectra. Although we give some results for the macro and mega climate regimes, we focus on weather, macroweather and climate: from dissipation to Milankovitch scales.

## Introduction

**Figure 1:**
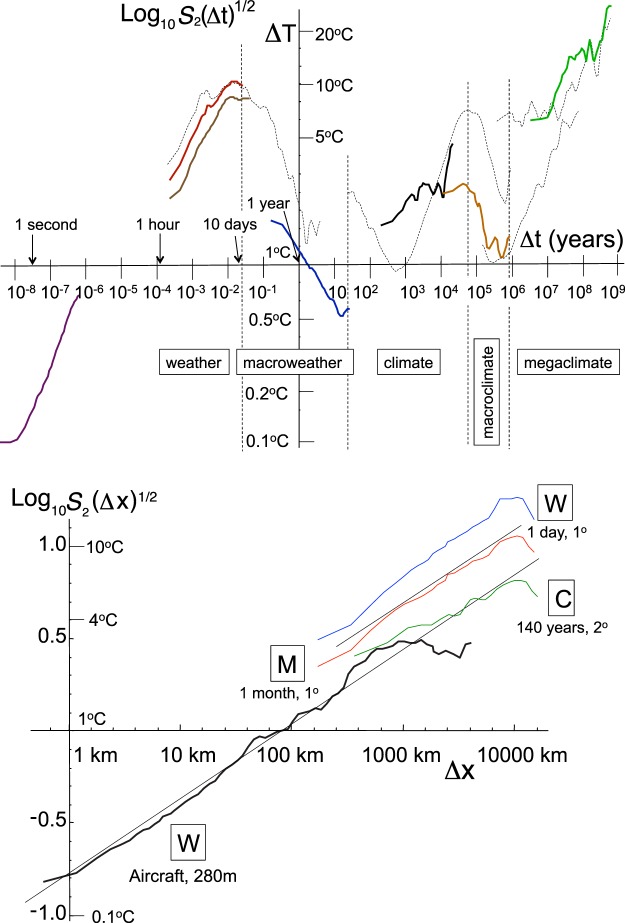
(a) (top panel): A wide scale range composite of temporal Root Mean Square (RMS) Haar temperature fluctuations with the data described in Table 1 in thick lines (from left to right, the solid curves are data set #1, bottom, #2, top #3), #4, #5, #7, #8). The dashed lines are reproduced from ref.^2^ left to right, datasets #2, #6, #7, #8. Here as in b, the fluctuations were multiplied by the canonical calibration constant of 2 so that when the slopes are positive, the fluctuations are close to difference fluctuations. The various scaling regimes are indicated at the bottom. (b) (bottom panel): Same as (**a**) but for spatial transects. The bottom aircraft (data set #9) and top (daily resolution, data set #10) data are in the weather regime (“W”), the line labelled “M” is in the macroweather regime (data set #11), and the one labelled (“C”), the climate regime (data set #12, see Table 1 for details). At scales >≈2500 km, the aircraft fluctuations from a single flight, have a dip caused by poor statistics. The breaks in the other (weather, macroweather and climate) transects only occur at scales of about 10,000 km (upper right).

The quasi-oscillatory model of atmospheric variability was first clearly articulated at the dawn of the paleo climate revolution by M. Mitchell^1^ who admitted that it was largely “an educated guess”. In spite of weak empirical support, it survived because even sophisticated analysis techniques were only applied over narrow ranges of scale so that the bigger picture was obscured. In addition, analyses were not sufficiently *simple*. Figure 1a spanning over 17 orders of magnitude in time (updated from^2^), clearly and simply shows how temperature and paleo temperatures vary with time scale; Fig. 1b shows examples of corresponding spatial statistics which are also scaling over large ranges.

New clarity was possible thanks to the rejuvenation of a fluctuation definition first proposed over a century ago^3^. For a time series *T*(*t*), the Haar fluctuation Δ*T*(Δ*t*) over a time scale Δ*t*, is simply the difference between the average of the first and second halves of the interval Δ*t*. Although technically, the Haar fluctuation is a wavelet, one doesn’t need any knowledge of wavelets to use them. The utility of the Haar fluctuation is not only that it is easy to define and implement numerically - even on series with missing data - but rather that its *interpretation* is simple. In regimes where the average fluctuations Δ*T*(Δ*t*) increase with scale Δ*t*, it is nearly the same as the difference *T*(*t*) − *T*(*t* − Δ*t*), in regimes where the average fluctuation decreases with scale, it is nearly the same as the anomaly – the average value of segments of length Δ*t* of a series whose mean has been removed^4^. The Haar fluctuations are adequate as long as the fluctuation exponent *H* is in the range −1 to 1 and this includes nearly all geophysical series (time) and transects (space; see below). With Haar fluctuations, wide scale range composites are both easy to produce and to interpret.

Yet, this is more than just new picture for classifying atmospheric variability. In this paper, we explore some of its consequences: extremes, sparseness, spurious spectral spikes, oscillations, outliers and tipping points.

## Spike Plots

**Figure 2:**
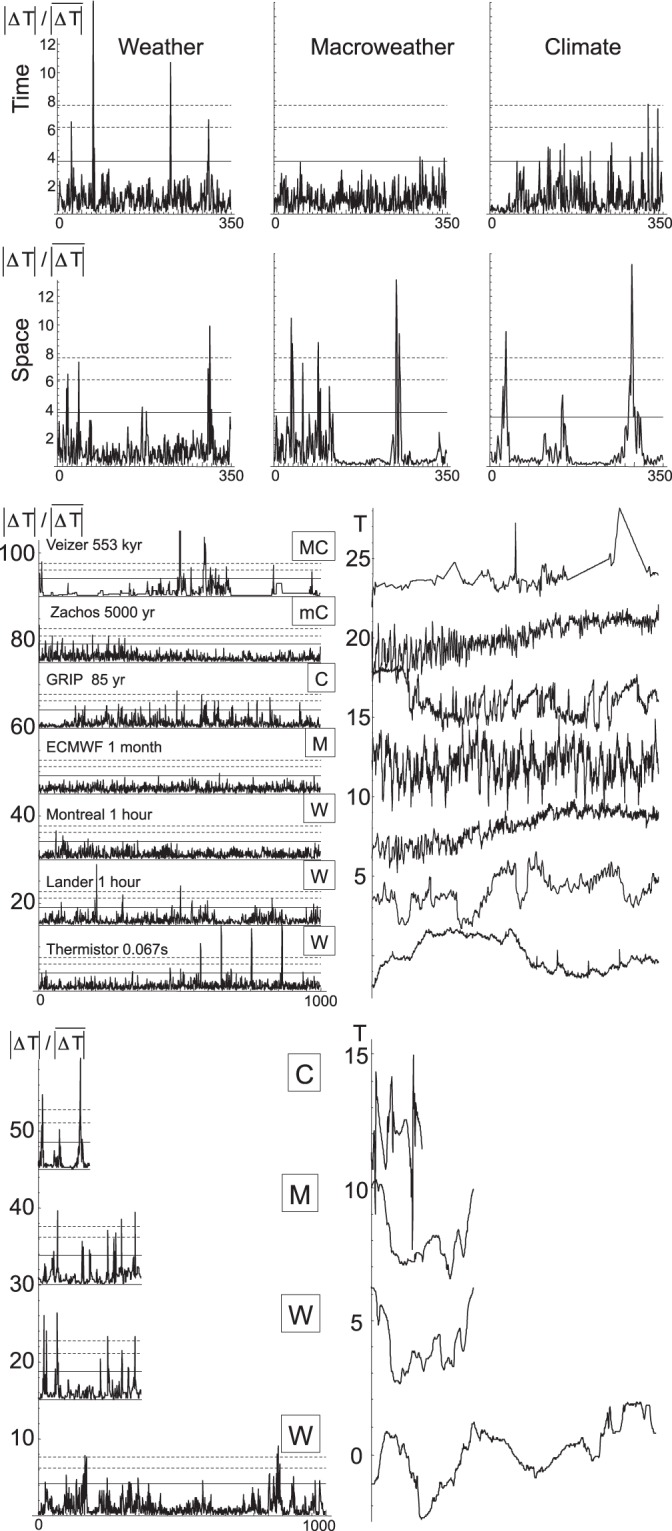
(a) (top panel): Temperature spike plots for weather, macroweather, climate (left to right) and time and space (top and bottom). The solid horizontal black line indicates the expected maximum for a Gaussian process with the same number of points (360 for each with the exception of the lower right which had only 180), the dashed lines are the corresponding Gaussian probability levels *p* = 10^−6^, *p* = 10^−9^, two of the spikes exceed 14; *p* < 10^−77^. The upper left is Montreal at 1 hour resolution (data set #3; these numbers refer to Table 1), upper middle, Montreal at 4 month resolution (#4), upper right, GRIP at 240 year resolution (#5), lower left, aircraft at 280 m resolution (#9), ECMWF, space, one month, 1° in space (#11), Twentieth Century Reanalysis (20 CR), 140 year res, 2° in space at 45°N, (#12). (**b**) (middle panel): Left: similar to (**a**) but for time only, and all series have 1000 points; the original series (normalized by their standard deviations and displaced vertically by 15 units for clarity) are shown on the right in the corresponding order. Bottom to top: The data are from sets #1,#2, #3, #4, #5, #7, #8 (Table 1). The “mC” and “MC” are for macro and mega climate respectively. The solid horizontal lines are the Gaussian levels corresponding to probability of 10^−3^, the dashed lines are for 10^−6^, 10^−9^. (c) (bottom panel): Same as (**a**) but for transects, bottom to top: length 1000 (aircraft, data #9), daily reanalysis (360 points, #10), monthly reanalysis, #11, 360 points, 140 year resolution, #12, 180 points.

In order to vividly display the extreme nonclassical behaviour, it suffices to introduce a seemingly trivial tool - a “spike plot”. A spike plot is the series (or transect) of the absolute first differences Δ*T* normalized by their means ($\overline{{\rm{\Delta }}T}$): ${\rm{\Delta }}T/\overline{{\rm{\Delta }}T}$, the overbar is the series or transect mean. Figure 2a shows examples for weather, macroweather and climate. The resolutions have been chosen to be within the corresponding regimes (see the details in Table 1). One immediately notices that with a single exception - macroweather in time – all of the regimes are highly “spiky”, exceeding the maximum expected for Gaussian processes by a large margin (the solid horizontal line). Indeed, for the other five plots, the maxima correspond to (Gaussian) probabilities *p* < 10^−9^ (the top dashed line), and four of the six to *p* < 10^−20^. The exception - macroweather in time - is the only process that is not very far from Gaussian behaviour, but it is nevertheless highly non Gaussian in space (bottom, middle). Physically, the spikes in the weather regime (left column) would correspond to meteorological fronts, whereas in the climate regime (upper right), they might correspond to Dansgaard-Oeschger events or (bottom right) to boundaries between different climate zones. More examples are shown in Fig. 2b,c, where we have included series both from high frequencies (showing “ramps”, bottom) as well as from the low frequency macro and mega climate regimes (top).

**Table 1:** A summary of the data used in this study, see Methods for more details.

	No.	Regime	Description	Resolution, time	Resolution, space
Time	1	weather	Thermistor, 2 hours	1/15 s	1 mm
	2	weather	Lander, 3 yrs	hourly	1 m
	3	weather	Montreal, 17 yrs	hourly	1 m
	4	macroweather	20 CR: 0–40 N, every 2° longitude	monthly	2° × 2°
	5	climate	GRIP paleo temp.	85 years	0.2 m
	6	climate	EPICA paleo temp.	30–700	0.55 m
	7	macroclimate	Zachos stack	5 kyrs	global
	8	megaclimate	Veizer stack	553 kyrs	global
Space	9	weather	Aircraft	0.5s	280 m
	10	weather	ECMWF reanalysis	daily	1°
	11	macroweather	ECMWF reanalysis	monthly	1° × 1°
	12	climate	20CR reanalysis	140 years	2° × 2°

In order to understand the spike plots, recall that if a process is scaling over time interval Δ*t* or spatial lag Δ*x*, fluctuations are generally of the form:

1 $$\begin{array}{cc}{\rm{\Delta }}T({\rm{\Delta }}t)={{\rm{\phi }}}_{{\rm{\Delta }}t}{\rm{\Delta }}{t}^{{H}_{t}}; & {\rm{\Delta }}T({\rm{\Delta }}x)={{\rm{\phi }}}_{{\rm{\Delta }}x}{\rm{\Delta }}{x}^{{H}_{x}}\end{array}$$

where φ is a dimensional quantity driving the process and the equality is understood in a statistical sense, the subscript indicates the resolution in time or in space. *H* is the fluctuation exponent; it is used in honour of Edwin Hurst who introduced it in hydrology^5^, but it is only the same as Hurst’s exponent for Gaussian processes; the fluctuation exponent is of more general utility. For example, in turbulence in space, if we replace *T* by a velocity component, and φ by the energy flux to the one third power, then *H* = 1/3 and the equation represents the Kolmogorov law. In general *H* (and the other scaling exponents) are different in space and in time and different in the horizontal and vertical directions. This is the problem of scaling stratification, scaling anisotropy, “Generalized Scale Invariance”^6^ it is out of our present scope; hence to lighten the notation we drop the *t*, *x* indices.

In the basic picture, φ is a Fourier space flux, in this example, the energy per time passing from small to large wavenumbers (large to small scales). The normalized spikes ${\rm{\Delta }}T/\overline{{\rm{\Delta }}T}$ can thus be interpreted as an estimate of the nondimensional, normalized driving flux:

2 $$\begin{array}{cc}{{\rm{\phi }}}_{{\rm{\Delta }}t}/\bar{{{\rm{\phi }}}_{{\rm{\Delta }}t}}={\rm{\Delta }}T({\rm{\Delta }}t)/\overline{{\rm{\Delta }}T({\rm{\Delta }}t)}; & {{\rm{\phi }}}_{{\rm{\Delta }}x}/\bar{{{\rm{\phi }}}_{{\rm{\Delta }}x}}={\rm{\Delta }}T({\rm{\Delta }}x)/\overline{{\rm{\Delta }}T({\rm{\Delta }}x)}\end{array}$$

The interpretation in terms of fluxes comes from turbulence theory and is routinely used to quantify turbulence (the physically significant flux may be some power of φ), but it turns out that for scaling processes, eq. 1 is of general validity. In the weather regime, in respectively time and space, the squares and cubes of the wind spikes are estimates of the turbulent energy fluxes. The spikiness is because most of the dynamically important events are sparse, hierarchically clustered (fractal), occurring mostly in storms and the centre of storms. The scaling in eq. 1 implies that the statistics of the fluctuations fall off in a (scale free) power law way. In comparison, classical autoregressive or moving average processes (time) or Kriging (space) involve much more rapid (exponential) decays of correlations. In contrast, scaling implies “long range” statistical dependencies, in series (time) they have “long range memories” (in Gaussian models, more restrictive definitions of long range memory are occasionally used that depend on the value of *H*).

The spikes could be considered as manifestations of “sudden transitions from quiescence to chaos”; a rather general definition of intermittency. Quantitative definitions applicable to fields (space) are of the “on-off” type, the idea being that when the temperature, wind or other field exceeds a threshold then it is “on” i.e. in a special state - perhaps of strong/violent activity. At a specific measurement resolution, the on-off intermittency can be defined as the fraction of space that the field is “on” (where it exceeds the threshold). In a scaling system, for any threshold the “on” region will be a fractal set and both the fraction and threshold will be characterized by exponents (*c* and by γ, eq. 4) that describe the intermittency over all scales and all intensities (thresholds). The spikes of each amplitude thus each have their own sparseness, and intermittency. In scaling time series, the same intermittency definition applies; note however that other definitions are sometimes used in series in deterministic chaos.

As long as *H* < 1 (true for nearly all geoprocesses), the differencing that yields the spikes acts as a high pass filter, the spikes are dominated by the high frequencies. Smoothed Gaussian white noises such as the scaling fractional Gaussian noise (fGn) and fractional Brownian motion (fBm) processes, or nonscaling processes such as autoregressive and moving average processes and the hybrid fractional versions of these, will have spikes that look like the macroweather spikes: in Fig. 2a, they will be roughly bounded by the solid horizontal lines. Mathematically, the scaling fGn, fBm are the results of power law filters (fractional integrals) of Gaussian white noises whereas more generally scaling processes are power law filters of densities of multifractal measures (the Fractionally Integrated Flux model^7^).

To go beyond Gaussians, each spike is considered to be a singularity of order γ:

3 $${\lambda }^{\gamma }=\frac{|{\rm{\Delta }}T|}{\overline{|{\rm{\Delta }}T|}}$$

λ is the scale ratio: λ = (the length of the series)/(the resolution of the series) = the number of pixels; in Fig. 2a,b, λ = 360, 1000 respectively. The extreme spikes ($|{\rm{\Delta }}T|/\overline{|{\rm{\Delta }}T|}$) in any 1000 point long series (e.g. Fig. 2b) have a probability ≈1/1000. For Gaussian processes, the spikes with this probability are $|{\rm{\Delta }}T|/\overline{|{\rm{\Delta }}T|}$ = 4.12, this is shown by the solid lines in Fig. 2b, the line therefore corresponds to ${\rm{\gamma }}=\,\mathrm{log}(|{\rm{\Delta }}T|/\overline{|{\rm{\Delta }}T|})/\mathrm{log}\,{\rm{\lambda }}\approx \,\mathrm{log}\,4.12/\,\mathrm{log}\,1000\approx 0.20$. For comparison, Table 2 gives the both the observed maximum γ for each series in Fig. 2 as well as the generally comparable theoretically expected maxima for the multifractal processes with the parameters estimated for the series in question.

**Table 2:** The scaling parameters *H*, *C*_1_, α and probability exponents *q*_*D*_.

	No.	*H*	*C*_1_	*α*	*q*_*D*_	*γ*_*max*_ (*theory*)	*γ*_*max*_ (*observed*)
Time	1	0.54 ± 0.003	0.013 ± 0.001	1.60 ± 0.08	3.1	0.34	0.39
	2	0.36 ± 0.02	0.011 ± 0.002	1.46 ± 0.05	3.4	0.31	0.38
	3	0.38 ± 0.01	0.021 ± 0.001	1.50 ± 0.07	6.2	0.22	0.27
	4	−0.24 ± 0.01	0.052 ± 0.003	1.56 ± 0.02	7.2	0.32	0.22
	5	0.20 ± 0.02	0.047 ± 0.006	1.40 ± 0.12	5.1	0.30	0.30
	6	0.41 ± 0.01	0.01 ± 0.01	1.46 ± 0.15	5.0	0.24	0.28
	7	−0.30 ± 0.03	0.083 ± 0.014	1.49 ± 0.13	3.3	0.44	0.31
	8	0.33 ± 0.03	0.107 ± 0.016	1.52 ± 0.31	1.7	0.65	0.52
Space	9	0.485 ± 0.004	0.055 ± 0.002	1.52 ± 0.16	3.5	0.38	0.38
	10	0.55 ± 0.02	0.070 ± 0.005	1.41 ± 0.06	13.0	0.35	0.38
	11	0.56 ± 0. 018	0.154 ± 0.006	1.55 ± 0.03	8.4	0.56	0.43
	12	0.47 ± 0.02	0.182 ± 0.011	1.64 ± 0.11	5.2	0.62	0.51

The far right columns give theoretical estimates of the maximum spike heights using the parameters *C*_1_, α, *q*_*D*_ and the scale ratio λ of the plots in Fig. 2 (=1000 except for data sets 10, 11, 12 where λ = 360, 360, 180 respectively; the spike plot for data set #6 is not shown). The theory column uses these parameters with the multifractal theory described in the text to estimate the solution to the equation *c*(γ_max_) = 1. The “observed” column determines γ_*max*_ from the spike plot directly: ${{\rm{\gamma }}}_{{\rm{\max }}}=\,\mathrm{log}(|{\rm{\Delta }}T|/\overline{|{\rm{\Delta }}T|}{)}_{{\rm{\max }}}/\mathrm{log}\,{\rm{\lambda }}$ where ${(|{\rm{\Delta }}T|/\overline{|{\rm{\Delta }}T|})}_{{\rm{\max }}}$is the maximum spike. For comparison, for λ = 1000, Gaussian probabilities of 10^−3^, 10^−6^, 10^−9^ yield respectively γ_*max*_ = 0.20, 0.26, 0.30. Error estimates for the right hand columns (extremes) were not given due to their sensitivity to the somewhat subjective choice of range over which the regressions were made.

In the general scaling case, the set of spikes that exceed a given threshold form a fractal set whose sparseness is quantified by the fractal codimension *c*(γ) = *D* − *d*(γ) where *D* is dimension of the space (*D* = 1 for series and transects) and *d*(γ) is the corresponding fractal dimension. The codimension is fundamental since it is the exponent of the probability distribution:

4 $$\begin{array}{cc}{\rm{\Pr }}(\frac{|{\rm{\Delta }}T|}{\overline{|{\rm{\Delta }}T|}} > s)\approx P(s){\lambda }^{-c({\rm{\gamma }})}; & {\rm{\gamma }}=\frac{\mathrm{log}\,s}{\mathrm{log}\,{\rm{\lambda }}}\end{array}$$

${\rm{\Pr }}(\frac{|{\rm{\Delta }}T|}{\overline{|{\rm{\Delta }}T|}} > s)$ is the probability that a randomly chosen spike $|{\rm{\Delta }}T|/\overline{|{\rm{\Delta }}T|}$ exceeds a fixed threshold *s, P*(*s*) is a nonscaling prefactor. *c*(γ) characterizes sparseness because it quantifies how the probabilities of spikes of different amplitudes change with resolution λ (for example when they are smoothed out by averaging). The larger *c*(γ), the sparser the set of spikes that exceed the threshold *s* = λ^γ^. A series is intermittent whenever it has spikes with *c* > 0.

Gaussian series are not intermittent since *c*(γ) = 0 for all the spikes. To see this, note that for Gaussian processes the cumulative probability of a spike exceeding a fixed threshold *s* is independent of the resolution λ, ${\rm{\Pr }}(|{\rm{\Delta }}T|/\overline{|{\rm{\Delta }}T|} > s)\approx P(s)$, where here, *P*(*s*) is related to the error function. Comparing this with Eq. 4, we see that *c* = 0.

Equation 4 is true for scaling series of any scale ratio λ (i.e. any length, any resolution); its characterization in terms of γ and *c*(γ) is independent of λ, scale invariant. Scale invariance is in fact the physical symmetry principle respected by the dynamics. Eq. 4 can be used to estimate the most extreme spike expected on a series with λ pixels. The probability of the corresponding singularity γ_*max*_ is ≈1/λ, hence from eq. 4, *c*(γ_*max*_) = 1 (equivalently, *d*(γ_*max*_) = 0) so that γ_*max*_ can be estimated from the inverse codimension function: γ_*max*_ = *c*^−1^(1); the far right column of Table 2 shows that this provides reasonable estimates.

Finally, we may note that *c*(γ) is not changed under certain transformations; for example under a linear change of variables. This means that although many of the analyses discussed here are proxy-based, as long as their calibration is linear, *c*(γ) and hence the intermittency will not be affected. In contrast, noise in the proxies (e.g. from bioturbation in ocean sediments, or isotope diffusion in ice cores) breaks the scaling at high frequencies; it will not affect the exponents estimated over the lower frequency scaling ranges. It is more difficult to generalize about the consequences of variable and uncertain chronologies, but at least in some cases - such as ice cores - the sampling intervals themselves can be highly variable – indeed spiky and scaling – yet at least in principle, the sampling intermittency may be statistically separated from the proxy intermittency (see box 11.3 in ref.^8^).

## Spectra

**Figure 3:**
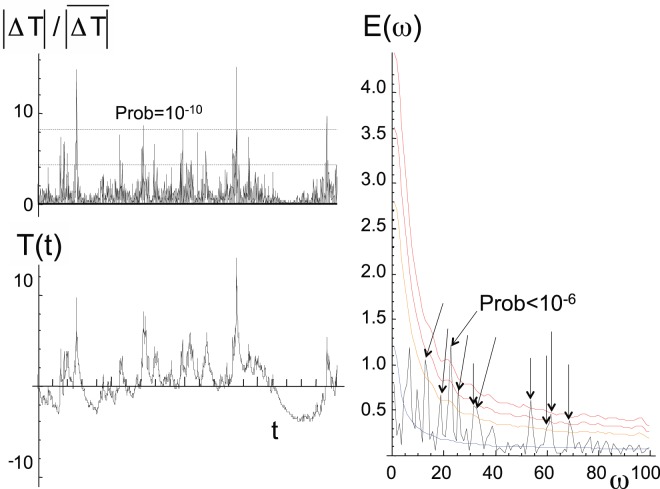
A multifractal simulation of a 2048 point series (lower left) with spike plot (upper left), and the corresponding spectrum (black, right). In the spike plot, the lower horizontal line corresponds to the maximum expected for a Gaussian process, the top dashed line indicates the 10^−10^ probability level. This realization was one of 5000 that were made with multifractal parameters *H* = 1/3, C_1_ = 0.05, α = 1.8 (see Methods and Table 1). The smooth blue curve is the ensemble average of the process (with theory and numerics superposed: they cannot be distinguished). Above the blue curve, is an orange 2 standard deviation curve, and (red), 3, 4 standard deviation curves (probabilities 0.1%, 0.003% respectively). The arrows indicate spikes with Gaussian *p* < 0.05.

Figure 2 shows that superficially routine series and transects can hide enormous nonclassical statistical variability. Until revealed by the spike plots, these fluctuations are virtually invisible yet they do not hide from spectral analysis which is highly sensitive to jumps. To explore this, we made a sample of 5000 realizations of a mildly intermittent multifractal process with parameters similar to those of the spiky regimes in Fig. 2, see Table 1 and Methods; here *H* = 0.33, *C*_1_ = 0.05, α = 1.6 and each realization had 2048 points. Figure 3 (bottom left) shows one of the simulations and the corresponding spike plot (top left) confirming that while the process itself is apparently only weakly variable (bottom left), it is nevertheless highly spiky and non-Gaussian with two of the spikes having Gaussian *p* ≪ 10^−9^. The spikiness is well detected by the spectral analysis which itself has spikes. The spectrum (the black curve) is of a single realization, it is a “periodogram” obtained with a standard Hanning window. The right panel of Fig. 3 reveals that with respect to the ensemble averaged spectrum (over 5000 periodograms from the same process, smooth, blue), nearly a dozen spectral spikes exceeded the conventional 2 standard deviation significance level (the arrows), with some exceeding the 4 standard deviation level (*p* ≈ 10^−5^). Although to make our point, we subjectively chose one of the more extreme of the first 50 simulations, this mild selection effect cannot explain the numerous strong spectral spikes. However, these spikes were to be expected since for each frequency, the tail of the probability distribution of the Fourier components was found to be a power law (here with exponent *q*_*D*_ ≈ 5, see below).

Imagine that we performed a spectral analysis, but didn’t know what to expect; e.g. an early analysis of ice core paleo temperatures. Encouraged by the idea that variability is synonymous with oscillations (e.g. the NOAA paleo-data website states that “because a particular phenomenon is called an oscillation, it does not necessarily mean there is a particular oscillator causing the pattern. Some prefer to refer to such processes as variability”, cited in ref.^2^), we might attempt to find a physical interpretation for some of the stronger spectral spikes. This temptation would be strengthened by the finding that some spikes are broad whereas (Gaussian) theory leads us to expect nearly independent Fourier components. Using sophisticated statistical methods such as multi-taper analysis (MTA) or singular spectral analysis (SSA), we could estimate the precise positions of the spectral spikes (see e.g. ref.^9^). With this, we could judge their statistical significance. The conventional null hypothesis is that the background residuals are autoregressive order 1 Gaussian processes. For many of the spikes in Fig. 3, we could reject this null hypothesis with very highly levels of confidence. For example, out the 100 lowest Fourier components shown, 10 would be significant at the 95% level (the arrows) and one would have a probability of less than 10^−6^. With a confidence level exceeding 99.9999%, we would be sorely tempted to postulate that we had uncovered strong evidence for the presence of a new quasi-periodic process.

It is tempting to conclude that many ephemeral (and presumably spurious) claims of oscillatory atmospheric (and other) processes might have been consequences of fast computers, Fast Fourier Transforms, combined with inappropriate null hypotheses.

## Understanding and Quantifying the Spikiness

**Figure 4:**
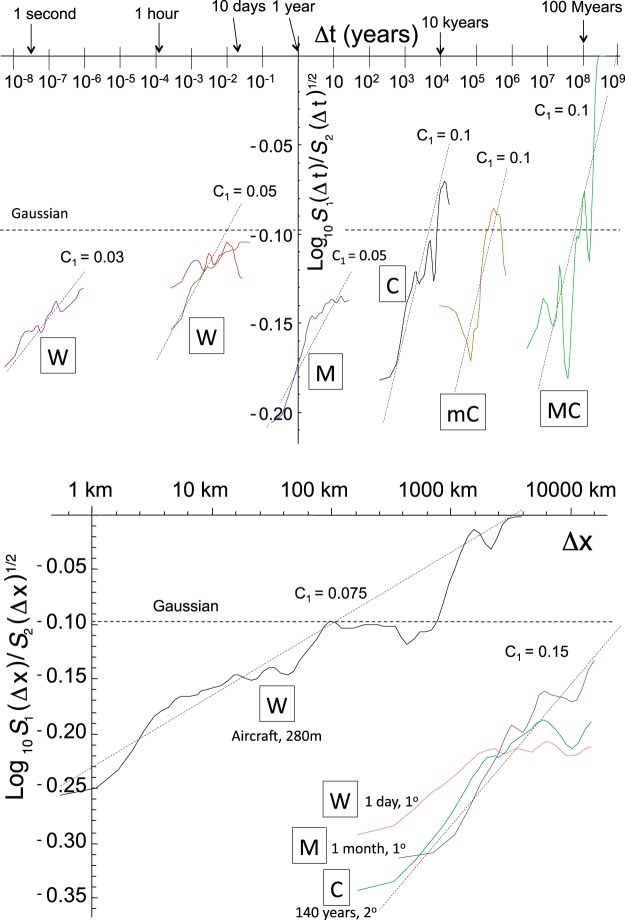
(a) (top panel): The ratios of the first moments (*S*_1_(Δ*t*)) to RMS moments (*S*_2_(Δ*t*)^1/2^) for the same data used in Fig. 2b. The slopes give the exponent *aC*_1_ (see the text). Using estimates of *a* = 0.86 (see Methods), reference lines with various intermittency exponents *C*_1_ are shown. Gaussian processes are independent of scale Δ*t*, they would lie along the dashed line. Since each curve is from *ratios* of statistics taken from data at different resolutions, we do not expect that the separate curves will be part of a single overall curve as they were in Fig. 1. (b) (bottom panel): The same as (**a**) but for the transects used in Fig. 2c.

In scaling processes, the variability systematically grows with scale range; this is the idea behind the cascade processes used in simulations (Fig. 3). Building up over a wide range of scales, the spikes exhibit clusters within clusters within clusters; we have seen how their sparseness can be quantified by fractal codimensions that decrease for stronger and stronger spikes. While it is possible to estimate *c*(γ) by directly analyzing the probabilities, it turns out to be advantageous to use mathematically equivalent, but technically simpler methods based on statistical moments. Although more details are given in Methods, the idea can be simply illustrated by plotting the ratio of the average absolute fluctuation to the root mean square fluctuation: the hierarchical clustering (i.e. clusters within clusters within clusters...) implies that this changes with scale in a power law manner: $\langle {\rm{\Delta }}T({\rm{\Delta }}t)\rangle /{\langle {\rm{\Delta }}T{({\rm{\Delta }}t)}^{2}\rangle }^{1/2}\propto {\rm{\Delta }}{t}^{a{C}_{1}}$ where *a* is a constant close to 1 (Methods) and *C*_1_ is the codimension of the mean, the basic intermittency exponent. The notation “<.>” denotes an average in the statistical (ensemble) sense. Figure 4a,b shows how the ratio changes with resolution for the different data sets. For Gaussian processes, $${{\rm{\sigma }}=\langle |{\rm{\Delta }}{\rm{T}}/\overline{{\rm{\Delta }}{\rm{T}}}|\rangle /{({\rm{\Delta }}{\rm{T}}/\overline{{\rm{\Delta }}{\rm{T}}})}^{2}\rangle }^{1/2}=\sqrt{\begin{array}{c}2\\ {\rm{\pi }}\end{array}}=0.798\ldots $$; it is a scale-independent constant indicated as a dashed line. Although more precise estimates of *C*_1_ are given in Table 1, the reference lines show that they are of the order of 0.03–0.10 in time and 0.075 to 0.15 in space (the ratios are noisy because of the large statistical fluctuations in each of the estimates of $\langle {\rm{\Delta }}T({\rm{\Delta }}t)\rangle$ and ${\langle {\rm{\Delta }}T{({\rm{\Delta }}t)}^{2}\rangle }^{1/2}$ that are amplified when the ratio is taken). The *C*_1_ values quantify how the intermittency near the mean builds up from one scale to another; even these apparently small *C*_1_ values can imply significant effects when λ is large. In addition, as shown in Methods their importance for moments grows rapidly with their order. For example, for the fourth order moment important for the kurtosis (the ratio of the fourth moment to the square of the second), the effective codimension is amplified by a factor of ≈9 (see Eq. M2 with *q* = 4, α = 1.6). In this case, even if the scaling only holds over a range of λ = 1000 and C_1_ = 0.1, this implies a kurtosis ≈36 compared to the Gaussian value 3.

## Extremes, Outliers, Black Swans and Tipping Points

The codimension *c*(γ) quantifies the *rate* at which – due to the clustering - the spike levels λ^γ^ build up as a function of the range of scales over which the dynamical processes act (eq. 3). *c*(γ) is nonlinear, requiring at least two parameters: the codimension near the mean (*C*_1_) and the multifractal index α that determines its curvature near the mean (eq. M3). However, it generally requires an additional third parameter *q*_*D*_ that characterizes the large γ behaviour, i.e. the tails of the probability distributions (eq. M8). This is because scaling in space and/or time generically gives rise to power law probability distributions (also known as “Pareto” or “fat-tailed” distributions). Specifically, the probability of a random temperature fluctuation Δ*T* exceeding a fixed threshold *s* is:

5 $$\begin{array}{cc}{\rm{\Pr }}({\rm{\Delta }}T > s)\approx {s}^{{q}_{D}}; & s\gg 1\end{array}$$

where *q*_*D*_ is a different exponent, this time characterizing the extremes.

**Figure 5:**
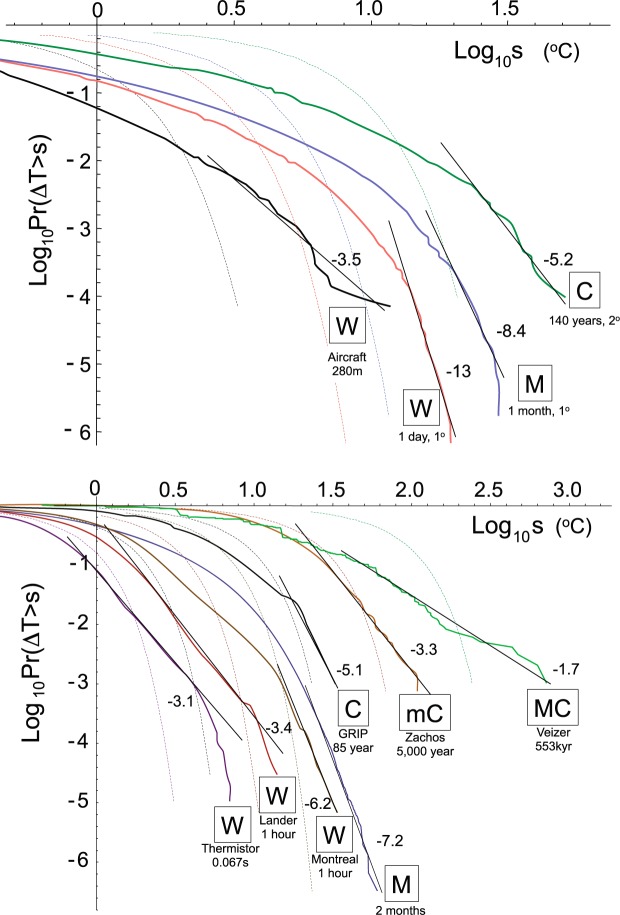
(a) (top panel): The probability distribution for absolute changes in the time series of Fig. 2b are shown. For clarity, they have been shifted to the right by factors left to right, respectively: 10, 1, 2, 4, 8, 16, 32). As discussed in Methods, reference lines give estimates of −*q*_*D*_ indicated in the figure and in Table 2. Also shown for reference (dashed curves) are the Gaussian distributions that give the same mean and standard deviations. (b) (bottom panel): The same as (**a**) but for the transects used in Fig. 2c. Note that there plausible arguments that numerical model outputs and reanalyses (the two middle curves) tend to be too smooth and to underestimate extremes; this might explain some of the larger *q*_*D*_ values (for weather and macroweather).

To get an idea of how extreme the extremes can be, consider an example with *q*_*D*_ = 5 (as has been estimated for the wind or temperature going back to ref.^10^ see Fig. 5 and Table 2). With this exponent, temperature fluctuations 10 times larger than typical fluctuations occur only 10^5^ times less frequently. In comparison, for a Gaussian, they would be ≈10^23^ times less likely; they would never be observed. Understanding the nature of the extremes is fundamental since it determines our interpretation of large events as either extreme - but nevertheless within the expected range, and hence “normal” - or outside this range, hence an “outlier” or perhaps even a “tipping point”.

A relevant example is global warming. Over about a century, there has been 1 °C warming of the globally averaged temperature - a roughly a 5 standard deviation event (with Gaussian probability ≈3 × 10^−6^). In spite of this, climate skeptics claim that it is no more than a Giant Natural Fluctuation (GNF) i.e. a change that might nevertheless be normal - albeit extreme. The relevant extreme centennial changes are indeed non-Gaussian, and bounding the probability tail between power laws with 4 < *q*_*D*_ < 6, ref.^11^ showed that the probability of extremes were enhanced by a factor of as much a factor of 1000. Yet the GNF hypothesis could nevertheless be statistically rejected with more than 99.9% confidence.

In the log-log plots shown in Fig. 5, we see that - as expected from eq. 5 - most of the distributions show evidence of log-log linearity near the extremes. When judging possible deviations, it could be recalled that due to inadequate instrumental response times, post-processing noise reduction procedures (e.g. smoothing) or via “outlier” elimination algorithms, that extremes can easily be underestimated. Also, several of the distributions in Fig. 5 are from reanalyses which are numerical model outputs. Since physically, the extremes are consequences of variability building up over a wide range of spatial scales, the models’ small hyperviscous scale range (truncated at ≈10^5^ m rather than at the viscous scale of ≈10^−3^ m), effectively truncates the extreme tails. Any of these effects could explain deviations from perfect power law tails or might explain some of the larger (i.e. less extreme) *q*_*D*_ values in Table 2. Finally, while power law probabilities arise naturally in scaling systems, the distributions are not *necessarily* power laws; non-power law (curved tails in Fig. 5) may simply correspond to the special cases where ${q}_{D}\to \infty$.

In any event, for each empirical distribution, Fig. 5 shows the Gaussian with the same mean and standard deviation (dashed). We see that the empirical distributions are generally quite far from Gaussian.

The power law fluctuations in Fig. 5a,b are so large that according to classical assumptions, they would be outliers. While Gaussians are mathematically convenient and can be justified when dealing with measurement errors, in atmospheric science thanks to the scaling, very few *processes* are Gaussian. Extremes occur much too frequently, a fact that colleagues and I regularly underscored starting in the 1980’s (for a review see Table 5.1a,b. in ref.^8^).

At best, Gaussians can be justified for additive processes, with the added restriction that the variance is finite. However, once this restriction is dropped, we obtain “Levy distributions” with power law extremes, but with exponents *q*_*D*_ < 2. The Gaussian assumption also fails for the additive but scaling *H* model^2,12^. Most importantly, Gaussians are irrelevant for multiplicative *processes*: these generally lead to power law extremes but without any restriction on the value of *q*_*D*_^7,13^. Related models include Self-Organized Criticality^14^ and Correlated Additive and Multiplicative noise^15^. Note that purely multiplicative random *variables* lead to somewhat less extreme log Levy and lognormal distributions (i.e. the logarithms are Levy or Gaussian; for the latter, see^16^ for an early review: their tails are “long” but not “fat”). The enhanced variability of multiplicative processes when compared to multiplicative variables is due to the singular small scale limit of the former; it has been theorized in the framework of multifractal phase transitions^17^. We could also mention that power law distributions also appear as the special (Frechet) case of Generalized Extreme Value Distributions although due to long range statistical dependencies, standard Extreme Value theory does not generally apply to scaling processes.

To underscore the importance of nonclassical extremes, Taleb^18^ introduced the terms “grey and black swans”. Originally, the former designated Levy extremes, and the latter was reserved for extremes that were so strong that they were outliers with respect to any existing theory. However, the term “grey swan” never stuck, and the better-known expression “black swan” is increasingly used for any power law extremes.

All of this is important in climate science where extreme events are often associated with tipping points. The existence of black swan extremes leads to a conundrum: since black swans already lead to exceptionally big extremes, how can we distinguish “mere” black swans from true tipping points?

## Discussion

The emerging picture of atmospheric dynamics is based on processes acting over wide ranges of scale occasionally interspersed with narrow scale range, quasi periodic processes, most notably the daily, annual and Milankovitch cycles. This picture not only clarifies the distinction between weather, macroweather and climate, it has implications for our understanding of spectra, intermittency and extremes. To make these obvious, we introduced spike plots that highlight hidden extreme jumps in series and transects, showing that the only regime plausibly compatible with Gaussian assumptions was macroweather in time. Unsurprisingly, Fourier analysis is sensitive to these jumps so that we expected (and found) random spikes in Fourier space (spectra) as well, potentially explaining numerous ephemeral claims of quasi-periodic behaviour.

The spikes have two features: they are clustered, occurring on sparse fractal sets, and their probability distributions have “fat” power law tails. Both features can be quantified by a codimension function *c*(γ) that quantifies how the sparseness changes with resolution, and is itself determined by three parameters. The first was quantified by estimating the exponent *C*_1_ from the variation of the ratio of the first and RMS moments, another (α) by the curvature of *c*(γ), and a final parameter (*q*_*D*_) from log-log plots of the probabilities of exceeding thresholds. We argued that in the atmosphere, strong intermittency and extreme black swan events are ubiquitous and that this has numerous consequences including for spectral analysis and for distinguishing “normal”, “expected” extremes from tipping points.

## Methods

### Data descriptions

The following are detailed descriptions of the data described in Table 1 and analyzed in the text. In Fig. 1a the solid lines are analyzed over the ranges indicated; the dashed lines are from the same sources but analyzed over wider scale ranges. The dashed and solid analyses of data # 2 differ lightly due to different daily and annual detrendings,

1. A series of 97,480 measurements from a thermistor (about 1 mm in size) that were taken at 15 Hz, from a location 0.44 m above the roof of the Physics building, McGill University, Montreal. The data was broken into 270 segments 24 s long.
2. Hourly temperature data from a station in Lander, USA (2006–2009). The data were detrended daily and annually and 144 sections (20 days each) were analyzed: 144 × 240 = 34,560 points in all.
3. Hourly temperature data from a station in Montreal, Canada (2000–2016). The data were detrended daily and annually and 419 sections (15 days each) were analyzed (419 × 15 × 24 = 150,840 points in all).
4. Monthly resolution data, from the 20^th^ Century Reanalysis (2° × 2°, monthly resolution) from 1871–2011 were used^19^. Data from 0 to 40 °N (21 latitude bands, 180 longitude bands) were broken into five 28 year segments (=336 months each), a total of 21 × 180 × 5 × 336 = 6,350,400 points. 28 years is roughly the longest possible without the low frequencies being dominated by anthropogenic warming.
5. Greenland GRIP paleo temperatures at 5 year resolution were degraded by factor of 17 (to 85 years) so as to have (roughly) pre-industrial climate regime resolution with a series 1032 points long (87,720 years). For analysis, this was broken into 20,000 year segments (the rough limit of the climate regime).
6. EPICA (Antarctica) ice core paleo temperatures; there were 5788 points spanning 801,000 years. In order to avoid highly nonuniform sampling, the *q*_*D*_ estimate was made from the equal depth data (55 cm resolution), see Fig. 5.21 in ref.^8^; the Haar analysis (shown as a dashed “S” shaped line in Fig. 1a) was performed using the offical chronology and a non interpolation algorithm^2^ and is reproduced from the latter.
7. A stack of benthic δ^18^O ratios^20^, with 14,825 points over the last 67 million years using the recommended −6.5 °C/mil calibration. The data from the first 6.85 million year were averaged to a 5,000 year resolutions (with only 3 missing data points being interpolated), 1,368 points in all. To avoid the larger scale mega-climate regime, this was then segmented into 8 segments each 855,000 years long to roughly represent the macroclimate period. The full length of the series was analyzed in ref.^2^, and is reproduced in Fig. 1a as a dashed line.
8. A stack of benthic δ^18^O ratios 2980 points^21^ very unevenly distributed over the Phanerozoic – back 553 million years. For the spike plot (Fig. 2b) and probability distribution (Fig. 5a), the range was broken into 1000 intervals (553 k year resolution) and the missing data was filled by linear interpolation. For the scaling exponents, the data was further degraded to 1.54 M year resolution to avoid the macroclimate regime at small scales (Fig. 1a which also shows the full analysis from ref.^2^, where it is indicated by a dashed line). The temperature calibration was the recommended value −4.5 °C/mil. This was the only series with significant interpolation (visible in Fig. 2b, top, right).
9. Data from an aircraft transect at 196 mb altitude (to within ±0.1%, about 12 km altitude) at 0.5 s resolution (the spatial resolution was 280 ± 2.8 m). This was from leg 1 of the Pacific Storms 2004 experiment (see ref.^22^), it had a total of 14,665 points covering a distance of 14,665 × 0.28 = 4102 km.
10. Data are from the European Centre for Medium Range Weather Forecasting (ECMWF) ERA-interim reanalyses at daily resolution for all the 31 days in January 2000 and from latitudes between 70°S and 70°N, 1° × 1° resolution. The spatial analysis was performed along the 141 lines of constant latitude for each of the days (141 × 31 = 4371 transects); each transect was over 360 points in longitude for a total of = 1,573,560 points.
11. Data are from ECMWF ERA-interim reanalyses at monthly and 1° × 1° resolution for the 12 months for 2000, from latitudes between 70°S and 70°N, resolution (12 months × 141 latitudes). The spatial analysis was over 360 longitudes for a total of 12 × 141 × 360 = 609,120 points.
12. Data are from the Twentieth Century Reanalysis (20 CR^19^) at monthly and 2° resolution, averaged over the period 1871–2012. All latitudes between ±60° were used (61 transects, each with 180 longitudes) for a total of 61 × 180 = 10980 points.

### Analysis techniques

The Haar fluctuation analyses (Fig. 1), the spike plots (Fig. 2), the spectral analysis (Fig. 3), the intermittency plot (Fig. 4) and the probability distributions (Fig. 5) are straightforward and were described in the main text. In this section, we indicate how the exponents (Table 2) were estimated.

In terms of fluctuations, we can conveniently express the generic scaling result by taking the *q* ^th^ order statistical moments of eq. 1:

M1 $$\begin{array}{cc}\langle {\rm{\Delta }}T{({\rm{\Delta }}t)}^{q}\rangle =\langle {{\rm{\phi }}}_{{\rm{\Delta }}t}^{q}\rangle {\rm{\Delta }}{t}^{qH}\propto {\rm{\Delta }}{t}^{{\rm{\xi }}(q)}; & {\rm{\xi }}(q)=qH-K(q)\end{array}$$

where $\langle {{\rm{\phi }}}_{{\rm{\Delta }}t}^{q}\rangle \propto {\rm{\Delta }}{t}^{-K(q)}$, and “<.>” is the ensemble average. $\langle {\rm{\Delta }}T{({\rm{\Delta }}t)}^{q}\rangle$is the (generalized) structure function and ξ(*q*) is its exponent (it is often convenient - e.g. eqs 3, 4 - to express the above in terms of the scale ratio λ. For series of length *T*, resolution Δ*t*, we have λ = *T*/Δ*t* so that λ ∝ 1/Δ*t*). *K*(*q*) is the exponent that characterizes how the statistics of the driving flux φ_Δ*t*_ change with scale Δ*t*; it directly characterizes the spikes (eq. 2). When φ_Δ*t*_is fairly constant - e.g. quasi Gaussian - then the various moments $\langle {\phi }_{{\rm{\Delta }}t}^{q}\rangle$ are independent of scale so that *K*(*q*) = 0 and the structure function exponent ξ(*q*) is linear (=*qH*, e.g. fGn or fBm). More generally, *K*(*q*) is a convex function (*K*″ > 0) with only constraints *K*(0) = 0 (for a nonzero process) and *K*(1) = 0 (the mean is independent of scale). A convenient characterization of the intermittency is thus *K*′(1) = *C*_1_, where *C*_1_ is the fractal co-dimension of the regions that give the dominant contribution to the mean of the process. More generally, due to the existence of stable, attractive, “universal” scaling processes:

M2 $$K(q)=\frac{{C}_{1}}{({\rm{\alpha }}-1)}({q}^{{\rm{\alpha }}}-q)$$

where 0 ≤ α ≤ 2 is the Levy index that characterizes the degree of multifractality^7^ since α = *K*″(1)/*K*′(1), it also characterizes the curvature of *K* near the mean (*q* = 1). The relationship in eq. M2 is only valid for *q* < *q*_*D*_ (below); for larger *q*, *K* diverges (note: this is only true for an infinite ensemble, for a finite ensemble, there will be “spurious scaling” and estimates of *K* will grow with the sample size).

We have already introduced the codimension function *c*(γ) that characterizes how the probability distributions change with scale ratio λ (eq. 4). Since a description in terms of probabilities is equivalent to one in terms of moments, it turns out that *c*(γ) and *K*(*q*) are related in a surprisingly simple way: by a Legendre transformation^23^. A consequence is that each order of moment is determined by a single level of spikes (singularities, γ). *C*_1_ is therefore the codimension of the set of spikes that dominates the calculation of the mean level of the spikes. The Legendre transformation of eq. M2 yields:

M3 $$c({\rm{\gamma }})={C}_{1}{(\frac{{\rm{\gamma }}}{{C}_{1}{\rm{\alpha }}^{\prime} }+\frac{1}{{\rm{\alpha }}})}^{{\rm{\alpha }}^{\prime} }$$

valid for γ > − C_1_α′/α otherwise = 0 and where the auxiliary variable α′ is defined by: 1/α′ + 1/α = 1. This means that processes whose moments have exponents *K*(*q*) given by eq. M2, have probabilities with exponents *c*(γ) given by eq. M3.

Since Gaussian processes are specified by their variances (the means of their signed fluctuations are zero), in Fig. 1a,b we have plotted the conventional the Root Mean Square fluctuation ${\langle {\rm{\Delta }}T{({\rm{\Delta }}t)}^{2}\rangle }^{1/2}\propto {\rm{\Delta }}{t}^{H-K(2)/2}$ (eq. M1) - rather than the scaling of the absolute mean $\langle {\rm{\Delta }}T({\rm{\Delta }}t)\rangle \propto {\rm{\Delta }}{t}^{H}$. For a Gaussian, *K*(2) = 0, the two would be the same; here they are different. In order to bring out this difference, and to quantify the intermittency which is responsible for it, in Fig. 4a,b we show the ratio $\langle {\rm{\Delta }}T({\rm{\Delta }}t)\rangle /{\langle {\rm{\Delta }}T{({\rm{\Delta }}t)}^{2}\rangle }^{1/2}\propto {\rm{\Delta }}{t}^{K(2)/2}$. In ref.^8^ we show how to construct a scaling function whose exponent yields *C*_1_ directly; however, since empirically, α ≈ 1.6 (see Table 1) the exponent ratio:

M4 $$a=\frac{(K(2)/2)}{{C}_{1}}=\frac{({2}^{{\rm{\alpha }}-1}-1)}{{\rm{\alpha }}-1}$$

varies in only a narrow range with α, (for α = 1.6, *a* = 0.86) in Fig. 4 we used this to plot various reference lines for *C*_1_. We see that *C*_1_ is larger in space than in time and is largest of all for the climate transects (Fig. 4b far right). This large intermittency is the statistical expression of the existence of various climate zones. For comparison, values of C_1_ in the range 0.05–0.10 are typical of turbulent fields; see Table 4.5 in the review ref.^8^.

In this paper, we estimated Δ*T*(Δ*t*) using Haar fluctuations; these were estimated by dividing the series and transects into disjoint intervals each of length Δ*t* or Δ*x* and taking the absolute difference of the first and second halves. Each interval yielded an estimate of a single fluctuation from which various powers were calculated. The corresponding statistical moments were estimated by averaging the powers over all the available fluctuations. For each order of magnitude of scale spanned by the data, 20 logarithmically spaced Δ*t*’s were chosen. The exponents ξ(*q*) were estimated by linear regression on a plot of the log(moments) versus logΔ*t*, and the uncertainties were estimated in the standard way. The use of more sophisticated regressions (and uncertainty estimation) techniques is not warranted since the values of ξ(*q*) depend on the somewhat subjective choice of scaling range used for their estimation. In addition, one would require the use of multifractal (rather than Gaussian) models for the underlying variability, and the corresponding statistical results are not yet available.

### Estimating α

The exponent α (eq. M2) is difficult to estimate since it characterizes how the nonlinear part of the structure function exponent ξ(*q*) varies with statistical moment *q*. Although various methods have been suggested, in Table 1 it was estimated by the following method. Following ref.^8^, section 11.2.3, we define:

M5 $$\begin{array}{cc}F({\rm{\Delta }}t)=\langle {\rm{\Delta }}T\rangle {(\frac{\langle {\rm{\Delta }}{T}^{1-{\rm{\Delta }}q}\rangle }{\langle {\rm{\Delta }}{T}^{1+{\rm{\Delta }}q}\rangle })}^{1/(2{\rm{\Delta }}q)}\approx {\rm{\Delta }}{t}^{{\rm{\xi }}(1)-{\rm{\xi }}^{\prime} (1)}\approx {\rm{\Delta }}{t}^{{C}_{1}}; & {\rm{\Delta }}q\ll 1\end{array}$$

(here we numerically took Δ*q* = 0.1). Then, following the discussion on intermittency, we defined the ratio:

M6 $$R({\rm{\Delta }}t)=\frac{\langle {\rm{\Delta }}T\rangle }{{\langle {\rm{\Delta }}{T}^{2}\rangle }^{1/2}}\approx {\rm{\Delta }}{t}^{{\rm{\xi }}(1)-{\rm{\xi }}(2)/2}\approx {\rm{\Delta }}{t}^{a{C}_{1}}$$

where *a* is defined in eq. S3. Finally, we estimate α from the ratio:

M7 $$a=\frac{\mathrm{log}\,R({\rm{\Delta }}t)}{\mathrm{log}\,F({\rm{\Delta }}t)}$$

which is independent of Δ*t* (uncertainties can be estimated from its small variations about a constant mean level). We can numerically invert eq. M4 to obtain α from knowledge of *a*.

### Estimating *q*_*D*_

We made histograms of absolute first differences of the series and transects using logarithmically spaced bins. We then accumulated these absolute changes starting with the largest to the smallest, yielding *Pr*(Δ*t* > *s*) for the probability that an absolute change Δ*t* exceeds the threshold *s* (note that the usual Cumulative Distribution Function (CDF) is *Pr*(Δ*t* < *s*) = 1 − *Pr*(Δ*t* > *s*)).

Figure 4a,b shows the resulting log-log plots whose slope is expected to asymptote to a constant value = −*q*_*D*_ (see eq. 5). The difficulty in the estimation is that the theory does not tell us the probability levels over which the power law is expected to hold and the value of *q*_*D*_ depends on which part of the probability distribution that the regressions are made. Once the power law region is identified, then an unbiased “Hill” estimator can be used to determine *q*_*D*_, (alternatively, the maximum likelihood method^24^) but the basic problem remains. In Fig. 5a,b, plausible reference lines were shown, and in Table 2, their slopes are given. For a review of several dozen relevant atmospheric *q*_*D*_ estimates, see Table 5.1a,b in ref.^8^.

In terms of *c*(γ), the extreme power law behaviour (eq. 5) corresponds to the linear large γ behaviour:

M8 $$\begin{array}{cc}c({\rm{\gamma }})=c({{\rm{\gamma }}}_{D})+({\rm{\gamma }}-{{\rm{\gamma }}}_{D}){q}_{D}; & {\rm{\gamma }} > {{\rm{\gamma }}}_{D}\end{array}$$

where γ_*D*_ is a critical singularity, given by the solution to the equation: *q*_*D*_ = c′(γ_*D*_). For universal multifractals, eq. M3 holds for γ < γ_*D*_ and so that *C*_1_, α, *q*_*D*_ completely determine *c*(γ), and hence (via eq. 4), the probabilities at all scales. Since, we showed that the expected maximum γ_*max*_ satisfies *c*(γ_*max*_) = 1, we can therefore theoretically calculate the maximum expected “spikes” in Fig. 2. The results are shown in Table 2 in the column γ_*max*_ (theory).

## Data Availability

All the data used are from publically accessible sources. The only exceptions are the thermistor data (#1) and the aircraft data (#9) that are available from the author on request.
